# Prospective Evaluation of Unprocessed Core Needle Biopsy DNA and RNA Yield from Lung, Liver, and Kidney Tumors: Implications for Cancer Genomics

**DOI:** 10.1155/2018/2898962

**Published:** 2018-12-10

**Authors:** Mikhail T. Silk, Nina Mikkilineni, Tarik C. Silk, Emily C. Zabor, Irina Ostrovnaya, Ari A. Hakimi, James J. Hsieh, Etay Ziv, Natasha Rekhtman, Stephen B. Solomon, Jeremy C. Durack

**Affiliations:** ^1^Interventional Radiology Section, Department of Radiology, Memorial Sloan Kettering Cancer Center, 1275 York Avenue, New York, NY 10065, USA; ^2^Department of Surgery, Urology Service, Memorial Sloan Kettering Cancer Center, 1275 York Avenue, New York, NY 10065, USA; ^3^Department of Epidemiology and Biostatistics, Memorial Sloan Kettering Cancer Center, 1275 York Avenue, New York, NY 10065, USA; ^4^Division of Oncology, Department of Medicine, Washington University Medical School, USA; ^5^Department of Pathology, Memorial Sloan Kettering Cancer Center, 1275 York Avenue, New York, NY 10065, USA

## Abstract

**Context:**

Targeted needle biopsies are increasingly performed for the genetic characterization of cancer. While the nucleic acid content of core needle biopsies after standard pathology processing (i.e., formalin fixation and paraffin embedding (FFPE)) has been previously reported, little is known about the potential yield for molecular analysis at the time of biopsy sample acquisition.

**Objectives:**

Our objective was to improve the understanding of DNA and RNA yields from commonly used core needle biopsy techniques prior to sample processing.

**Methods:**

We performed 552 ex vivo 18 and 20G core biopsies in the lungs, liver, and kidneys. DNA and RNA were extracted from fresh-frozen core samples and quantified for statistical comparisons based on needle gauge, biopsy site, and tissue type.

**Results:**

Median tumor DNA yields from all 18G and 20G samples were 5880 ng and 2710 ng, respectively. Median tumor RNA yields from all 18G and 20G samples were 1100 ng and 230 ng, respectively. A wide range of DNA and RNA quantities (1060–13,390 ng and 370–6280 ng, respectively) were acquired. Median DNA and RNA yields from 18G needles were significantly greater than those from 20G needles across all organs (*p* < 0.001).

**Conclusions:**

Core needle biopsy techniques for cancer diagnostics yield a broad range of DNA and RNA for molecular pathology, though quantities are greater than what has been reported for FFPE processed material. Since non-formalin-fixed DNA is advantageous for molecular studies, workflows that optimize core needle biopsy yield for molecular characterization should be explored.

## 1. Introduction

Image-guided solid tumor needle biopsies are frequently the starting point for modern cancer care. The ability to genomically characterize tumors has amplified the importance of tissue biopsies for cancer treatment selection, determining eligibility for clinical trials and understanding disease progression. In recent years, the brisk pace of discoveries revealing the genetic basis for malignant transformation has empowered oncologists, enabling therapies targeting specific molecular aberrations [1–3]. Needle biopsies can provide material for targeted genetic mutation analysis or to assess response to treatment, obviating the need for surgical biopsy.

A high-quality, high-value biopsy is now defined by sufficient cancer cellularity for diagnosis and genomic analysis [4]. Diagnostic rates for contemporary targeted biopsies are high, but procedural practice guidelines have been slow to consider additional sampling requirements associated with molecular characterization [5]. Real-time CT, ultrasound, or MR image-guidance technologies have enabled more accurate percutaneous sampling of smaller targets [6]. However, the quantity of genetic material that can be obtained from small tumors is not easily defined due to many factors influencing biopsy yield, including normal tissue versus solid tumor cellularity and variable density of tumor nuclei per volume of tissue. Furthermore, single-site biopsies may not sufficiently portray intratumoral genetic heterogeneity [7].

Deoxyribonucleic acid (DNA) and ribonucleic acid (RNA) quantities required for a combination of routine clinical care, clinical trials, and research protocols often vary by individual institution and clinical team. Quantities sufficient for analysis will also vary in relation to the increasing number and range of molecular tests and technical advances in tissue analytics. Furthermore, several analyses of preanalytic factors related to tumor sequencing have raised concerns about low DNA and RNA yields from percutaneous tumor biopsies [8, 9].

Importantly, standard core biopsy processing in pathology laboratories includes formalin fixation and paraffin embedding (FFPE). All downstream diagnostic and molecular assays are generally performed on thin sections prepared by microtomy from FFPE tissue blocks. In most studies to date, DNA and RNA content in core biopsies has been analyzed from FFPE material, whereas quantities of nucleic acid in unprocessed core biopsies are not well established. The goal of this study was to assess DNA and RNA quantities obtained using widely used core biopsy techniques from different cancer types in order to facilitate planning and decision-making with regard to molecular oncology testing. Knowledge of needle biopsy sampling capabilities can be essential for patient management in the setting of either known or suspected cancer. For both patient and healthcare provider, the anticipated value of quantitative data to plan needle biopsies is a better understanding of the potential risk versus clinical benefit [10–12].

## 2. Materials and Methods

We performed an Institutional Review Board-approved prospective study of surgically resected specimens at a comprehensive cancer center with a waiver of informed consent. Biopsies were performed in a tissue procurement service facility under direct visualization within 2 hours of surgical excision using 18-gauge (18G) and 20-gauge (20G) core biopsy needles (Temno Evolution, CareFusion, Waukegan, IL). Each surgical specimen was first dissected to allow direct visualization of the tumor and surrounding normal tissues. Biopsies were acquired from a variety of locations in normal parenchyma and tumor, avoiding areas of visible necrosis, and each 2 cm long core needle sampling tray was visually inspected. Core specimens that did not fill at least 85% of the sampling tray were discarded. Biopsies were performed in triplicate using 18G and 20G needles for both DNA and RNA processing. Biopsy sample sizes were estimated based on the number of samples required to achieve statistical significance from a preliminary kidney biopsy cohort. Each specimen was immediately placed in a 1.7 ml Eppendorf tube and snap frozen in liquid nitrogen. Samples were then stored in a −80°C freezer until molecular extractions were performed.

### 2.1. DNA Extraction

DNA was extracted using a standard protocol (DNeasy, Qiagen, Venlo, Netherlands) with 4 *μ*l RNase A added immediately after incubation. 50 *μ*l of 10 nM Tris-Cl and 0.5 mM EDTA buffer (AE, pH 9.0) were used for the elution step.

### 2.2. RNA Extraction

RNA was extracted in an RNase-free environment according to the standard product protocol (RNeasy, Qiagen). All RNA samples were kept on dry ice during extraction. Tissues were lysed using 1.4 mm ceramic spheres (lysing matrix D, MP Biomedicals, Solon, OH) in a tissue homogenizer (Fast Prep 24, MP Biomedicals) and 650 *μ*l of lysis buffer (RLT Buffer, Qiagen) with the addition of on-column DNase digestion before RNA purification. 30 *μ*l of RNase-free water was used to elute all samples.

### 2.3. Quantitative Measurements

DNA and RNA quantity (total DNA and RNA) was calculated from concentration multiplied by volume. Concentration was measured using a spectrophotometer (Nanodrop 2000, Thermo Scientific). If the measured ratio of absorbance at 260 : 280 was less than 1.6 for DNA or 1.8 for RNA, the samples were run for an additional time on the chromatography columns in the extraction protocol until the purity threshold was reached.

### 2.4. Statistical Analysis

The three repeated observations for each tumor sample were averaged into a single observation for analysis after examining the variation of repeated observations using descriptive statistics and graphical displays. Box plots of averaged data were generated for each tumor separately for RNA and DNA and by needle gauge (18G versus 20G) and tissue type (normal vs. tumor). For comparisons between tissue type and needle gauge, the Wilcoxon signed-rank test for paired data was used. For comparisons across organ sites (lung versus liver versus kidney), the Kruskal-Wallis rank sum test was applied. A *p* value < 0.05 was considered statistically significant. Analyses were conducted using R software version 3.1.0 (R Core Development Team, Vienna, Austria).

### 2.5. Results

A total of 552 ex vivo biopsies from 46 surgically resected lung (*n* = 15), liver (*n* = 15), and kidney (*n* = 16) specimens were performed.  Table 1 indicates the number of biopsies obtained from each organ and the final pathologic diagnosis for each tumor type. The quantitative yield by organ, needle gauge, and tissue type (normal vs. tumor) is provided for DNA and RNA in Tables 2 and 3, respectively.

### 2.6. DNA Yield

**Figure 1:**
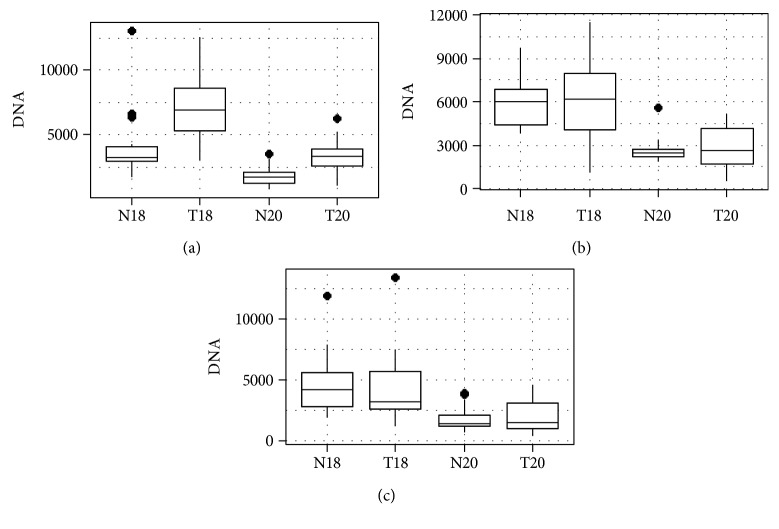
DNA content by tissue type and needle gauge in (a) lung tumors, (b) liver tumors, and (c) kidney tumors (N18 = normal tissue/18 gauge, T18 = tumor tissue/18 gauge, N20 = normal tissue/20 gauge, and T20 = tumor tissues/20 gauge). The dark bar represents median DNA quantity, the surrounding box encompasses the 25–75% interquartile range (IQR), and the brackets reflect 1.5^∗^IQR. Diamonds represent statistical outliers.

For all pooled organ sites, the median DNA yield from the larger 18G biopsy needles was significantly greater (*p* < 0.001) than that from 20G needles in both tumor and normal tissue samples. Median DNA quantities were greater for lung tumor samples compared to normal lung tissue (18G biopsies, *p* < 0.001; 20G biopsies, *p* < 0.001). There was no statistical difference in median DNA obtained from normal versus tumor tissues in the liver or kidney. For all cancer types sampled, the median DNA quantity acquired from single-needle pass 18G and 20G core biopsies was 5880 ng (range 1060–13390 ng) and 2710 ng (range 370–6280 ng), respectively. Box plots in Figure 1 depict median DNA content as well as interquartile ranges for each tissue type and biopsy needle gauge.

### 2.7. RNA Yield

**Figure 2:**
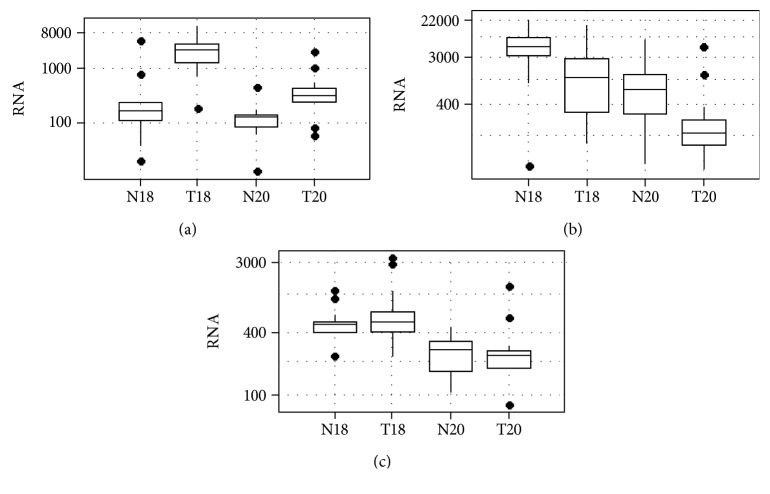
Logarithmic scale of RNA content by tissue type and needle gauge in (a) lung tumors, (b) liver tumors, and (c) kidney tumors (N18 = normal tissue/18 gauge, T18 = tumor tissue/18 gauge, N20 = normal tissue/20 gauge, T20 = tumor tissues/20 gauge). The dark bar is the median, the box encompasses the 25–75 interquartile range (IQR), the dotted brackets are 1.5^∗^IQR, and dots are outliers.

The median RNA yield from 18G needles was also significantly greater (*p* < 0.001) than that from 20G biopsies when tumor and normal samples were pooled for all organs. Median RNA quantities were greater for lung tumor tissue compared to nonmalignant tissues for 18G and 20G from the lungs (18G biopsies (*p* = 0.001) and 20G biopsies (*p* < 0.001), respectively) and liver (*p* = 0.012 and *p* = 0.002, respectively), but not from the kidney. The median RNA quantity from 18G and 20G cancer biopsies was 1100 ng (range 110–17210 ng) and 230 ng (range 60–5210 ng), respectively. Box plots in Figure 2 depict median RNA quantities and interquartile range by needle gauge and tissue type.

## 3. Discussion

In the recent years, cancer genetic technologies such as next-generation sequencing (NGS) have evolved, offering insights beyond traditional histopathologic or radiographic diagnoses [13]. Increased emphasis on molecular characterization has highlighted the role of targeted tissue biopsies in oncology, now routinely obtained for personalized treatment planning and for correlative studies in clinical trials. Gene sequencing for mutation profiling can be particularly challenging for solid tumors as formalin fixatives can disrupt DNA integrity [14]. As nucleic acid yield is not enumerated at the time of biopsy, even when on-site cytopathology review is performed, it can be difficult to determine whether sufficient genetic material has been obtained [15].

While tumor heterogeneity, cellularity, and size as well as other preanalytic parameters and factors can impact downstream analytic success, important information can be gained from studies examining DNA and RNA yield using standardized ex vivo conditions [16, 17]. Notably, one previous study focused on lung tumor core biopsies reported no statistical difference between in vivo and ex vivo nuclei acid yields within cohorts of the same tumor type [17]. These same authors also attempted to predict tissue yields from core biopsies using needles of different gauges used in clinical practice with multivariate regression. A moderately strong correlation between calculated sampling volume and nucleic acid yield was observed, though analysis was limited to lung tumors and a relatively small number of biopsy samples. In this study, we examined a larger number of primary and metastatic tumor biopsies from the lungs, livers, and kidneys, increasing the potential generalizability of our findings.

Only in the lung, and not in the liver or kidney, did we observe a statistically significant difference in DNA quantities obtained from normal parenchyma versus tumor tissues. In this case, increased cell density, particularly relative to normally air-filled lung tissues, and higher nuclear to cytoplasmic ratios may account for higher quantities of genetic material in lung tumor samples versus normal aerated lung [18, 19]. RNA differences were observed in the liver and lung but not observed in the kidney. A previous study also reported no difference in RNA content between primary renal malignancies and normal renal parenchyma [20].

Similar to other studies, we found that larger 18G needles acquired twofold more DNA and fivefold more RNA on average compared to 20G needles, suggesting that additional needle passes may be necessary to obtain sufficient genetic material when using smaller-gauge needles. The clinical implications of substantial yield variance should not be minimized however, as linear models have not been validated in clinical practice, and smaller 20G needles can effectively reveal clinically meaningful mutations in lung tumors [17, 21]. Based on our findings, the notion that one additional large volume core needle pass will guarantee nucleic acid sampling adequacy could lead to analytic failure. In real-world practice, percutaneous biopsy indications, approach, and technique must be considered to minimize procedural morbidity and maximize efficacy. In a recent meta-analysis, the risk for complications following a lung biopsy correlated with larger biopsy needles [22]. Prior knowledge of minimum sampling requirements can facilitate estimation of biopsy feasibility, safety, and likelihood of success. In particular, the number and type of analytic studies to be performed can influence biopsy decisions as higher complication rates are associated with increased needle passes and larger-gauge needles [10, 11]. Ideally, specimen quantities would be well balanced with procedure time and the lowest achievable patient risk.

The practical implications of this study are most apparent in relation to contemporary genetic testing requirements and sources of preanalytic biopsy sample variation. Minimum DNA for NGS can vary depending upon the clinical laboratory technology platform, as well as the target enrichment strategy and number of genes tested in a panel. For example, for the NGS platform currently used at our institution—hybridization capture MiSeq Illumina-based MSK-IMPACT assay [23]—200–250 ng of DNA is optimally required. DNA quantities as low as 10 ng may be successfully analyzed using the Ion Torrent (Thermo Scientific, Waltham, MA) platform [8], though sequencing errors may occur with limited DNA.

Biopsy sample RNA profiling is highly dependent upon quality, as fragmentation or degradation by RNases can hamper mutational analysis. Microarray technologies can be used to analyze 500 ng of RNA, while NGS and amplification techniques can substantially lower thresholds for clinically meaningful sequencing, even to the level of single-cell genetic material [24, 25].

Assuming no degradation, fragmentation, or other preanalytic disruption of biopsy samples, based on the median DNA content revealed in this study, a single 2 cm long 18G or 20G biopsy should be sufficient for most contemporary NGS assays. The same holds true for RNA; however, at the lower end of the RNA range, as low as 60 ng for 20G core needle biopsies, a single biopsy sample may be more susceptible to analytic failure. In practice, sample degradation and disruption do occur during acquisition and processing. The routine fixation methods fail to conserve the structure of nucleic acids and proteins in tissues. Even short-term treatment of sections with formalin has been shown to significantly reduce the DNA solubility. Similarly, the extraction of useful RNA from FFPE tissue is often compromised because of incomplete lysis leading to poor extraction efficiency. In a recent study, RNA extracted from FFPE samples was severely degraded compared to fresh-frozen samples [26]. In our results, the DNA and RNA yield prior to sample processing was 4–6-fold greater than what has previously been reported for FFPE cell blocks [27]. Our results reveal unprocessed nucleic acid yield from core biopsies, which can be frozen at the point of acquisition and submitted for DNA sequencing without FFPE. The potential limitation of this approach is that direct molecular characterization without histopathologic confirmation could result in unconstructive processing of nontumor tissues. Therefore, workflows that increase tumor yield from biopsies, such as radiographic image guidance to confirm needle position within tumor tissues, could mitigate this limitation. Ongoing work suggests that transmission optical spectroscopy imaging of fresh core samples can rapidly characterize tissues at the point of acquisition, which could be used to select appropriate samples for flash freezing prior to molecular diagnostic assays [28].

Although the biopsy yields reported here can serve as a reference for physicians planning or performing molecular studies, the following limitations must be considered in regard to the generalizability of our data. Many factors may reduce the quantity of RNA and DNA suitable for analysis within a small solid tumor biopsy sample, including prior chemotherapy [29] or tumor-associated desmoplasia [30, 31]. In addition, necrotic tissue has a lower cellular content and can adversely impact biopsy efficacy, even when molecular studies are not planned [32, 33]. While each biopsy was visually screened for a minimum tissue sample length, we did not examine tissues at the microscopic level for cellular composition. We selected tumors that were large enough to fill a 2 cm core needle biopsy sampling tray. As increased tumor size is associated with increased necrosis [34], we cannot be certain that molecular quantities reported here are valid for larger tumors. By the same token, we cannot certify based on these data that tumors smaller than 2 cm will contain less genetic material in linear proportion to biopsy sample length. We also used common spectroscopy-based techniques to quantify the molecular content of biopsy samples; however, these types of measurements can result in overestimations. Decreased accuracy is attributed to poor 260 : 280 nm absorption ratios and cross-contamination of RNA and DNA [35]. We used recommended extraction protocols to remove DNA or RNA contaminants; however, remaining contaminants could have influenced yield. Finally, due to the wide variation in the operator technique impacting sampling volumes, we did not study common alternative needle biopsy methods such as fine-needle aspiration.

In summary, we report a wide range of nucleic acid quantities obtained from core needle biopsies in organs commonly afflicted with primary or metastatic cancer. Overall, unprocessed sample nucleic acid quantities are increased relative to FFPE processed tissues; therefore, workflows that bypass fixation and paraffin-based processing may improve yield and utility of core needle sampling for molecular diagnostics.

## Figures and Tables

**Table 1:** Pathologic tissue diagnoses by organ.

*Kidney ( n* = 16* )*	
Clear cell carcinoma	13
Papillary carcinoma	1
Unclassified renal cell carcinoma	1
Chromophobe carcinoma	1
*Liver ( n* = 15* )*	
Colorectal adenocarcinoma	12
Hepatocellular carcinoma	1
Cholangiocarcinoma	1
Lung adenocarcinoma	1
*Lung ( n* = 15* )*	
Squamous cell carcinoma	6
Lung adenocarcinoma	5
Carcinoid	1
Metastatic poorly differentiated carcinoma	1
Mucinous carcinoma	1
Lymphoma	1
*Total number of specimens biopsied*	**46**
*Total biopsy samples (normal + tumor tissues)*	**552**

**Table 2:** Median DNA (range) from 18- versus 20-gauge needle samples from normal and tumor tissues obtained from the kidney, lung, and liver.

	18G biopsy (ng DNA)	20G biopsy (ng DNA)	*p* value (18G vs. 20G)
Any organ			
Normal	4350 (1730, 13040)	1970 (700, 5620)	*<0.001*
Tumor	5880 (1060, 13390)	2710 (370, 6280)	*<0.001*
Kidney			
Normal	4150 (1930, 11890)	1360 (700, 3870)	*<0.001*
Tumor	3170 (1180, 13390)	1450 (370, 4600)	*<0.001*
*p* value (normal vs. tumor)	1.00	0.890	
Lung			
Normal	3240 (1740, 13040)	1720 (760, 3520)	*<0.001*
Tumor	6910 (3070, 12570)	3350 (1110, 6280)	*<0.001*
*p* value (normal vs. tumor)	*<0.001*	*<0.001*	
Liver			
Normal	6050 (3790, 9740)	2480 (1890, 5620)	*<0.001*
Tumor	6190 (1060, 11530)	2630 (480, 5160)	*<0.001*
*p* value (normal vs. tumor)	0.804	0.847	

**Table 3:** Median RNA (range) from 18- versus 20-gauge needle samples from normal and tumor tissues obtained from the kidney, lung, and liver.

	18G biopsy (ng RNA)	20G biopsy (ng RNA)	*p* value (18G vs. 20G)
Any organ			
Normal	510 (30, 23540)	240 (30, 7090)	*<0.001*
Tumor	1100 (110, 17210)	230 (60, 5210)	*<0.001^a^*
Kidney			
Normal	480 (230, 1210)	270 (110, 460)	*<0.001*
Tumor	510 (220, 3420)	290 (70, 2480)	*<0.001*
*p* value (normal vs. tumor)	0.855	0.217	
Lung			
Normal	150 (30, 4940)	*120 (30, 400)*	*0.008*
Tumor	2870 (170, 12700)	290 (70, 2480)	*<0.001*
*p* value (normal vs. tumor)	*<0.001*	*<0.001*	
Liver			
Normal	4740 (60, 23540)	700 (60, 7090)	*<0.001*
Tumor	1190 (110, 17210)	150 (60, 5210)	*<0.001*
*p* value (normal vs. tumor)	*0.012*	*0.002*	

^a^Exact test could not be performed due to ties, normal approximation used.

## Data Availability

The data used to support the findings of this study are available from the corresponding author upon request.
